# Perceptions of aquatic physiotherapy and health‐related quality of life among people with Parkinson’s disease

**DOI:** 10.1111/hex.13202

**Published:** 2021-02-16

**Authors:** Aan Fleur Terrens, Sze‐Ee Soh, Prue Morgan

**Affiliations:** ^1^ Movement Disorder Program Peninsula Health Frankston VIC Australia; ^2^ Department of Physiotherapy Monash University Frankston VIC Australia; ^3^ Department of Epidemiology and Preventative Medicine Monash University Frankston VIC Australia

**Keywords:** aquatic physiotherapy, hydrotherapy, Parkinson's disease, patient perspectives, quality of life

## Abstract

**Background:**

Enablers for people with Parkinson's disease (PD) participating in aquatic physiotherapy have been identified, and exercise improves health‐related quality of life (HRQoL) but it is unclear whether all enablers and barriers for aquatic physiotherapy specific to the PD population have been explored.

**Objective:**

To describe HRQoL in people with PD who have undertaken aquatic physiotherapy, and explore their perceptions and attitudes regarding the programme.

**Methods:**

Twenty‐one participants who participated in a pilot trial on aquatic physiotherapy were included. Participants completed a survey regarding their experiences. The Parkinson's Disease Questionnaire‐39 (PDQ‐39) and Personal Well‐being Index‐Adult (PWI) were used to quantify HRQoL, whilst focus groups were conducted to explore their perceptions and attitudes. Descriptive statistics were used to summarize HRQoL scores. Focus group data were analysed using the deductive coding method.

**Results:**

Most participants felt that the aquatic programme was worthwhile (n = 20/21, 95%). However, they had poor overall well‐being (mean 41.6, SD 13.5) and HRQoL (mean 31.0, SD 13.2) as measured by the PWI and PDQ‐39. Several barriers to aquatic therapy including safety when getting dressed, fatigue and transport were identified although many enablers were also identified, including an improvement in function, less falls and group socialization.

**Conclusions:**

Aquatic physiotherapy was well‐accepted. Participants felt their function improved and felt safe in the water. HRQoL is lower in individuals with PD when compared to Australian norms; thus, interventions to optimize HRQoL need to be explored further.

**Patient or Public Contribution:**

Patients participated in the aquatic intervention, survey and focus groups.

## 1 INTRODUCTION

Parkinson's disease (PD) is a progressive neurological disorder that has a significant impact on both motor function and health‐related quality of life (HRQoL).^1^, ^2^ The ability to communicate and have social connectedness has been associated with better HRQoL outcomes in people with PD,^3^ whilst depression and the incidence of falls has been found to be a strong predictor of low HRQoL scores in this population.^4^, ^5^, ^6^, ^7^ As falls occur in 30%‐50% of all people with PD,^8^ it is vital to find treatment options that can improve both postural control and HRQoL. Although exercise has been seen to have a positive effect on falls and HRQoL,^9^ research has indicated that common barriers such as lack of time, fear of falling and low outcome expectation are perceived in community dwelling individuals with PD.^9^ Previous research has also shown that participation in exercise programmes is low in people with PD.^10^, ^11^, ^12^

Evidence suggests that aquatic physiotherapy may have a positive effect on HRQoL in people with neurological conditions such as multiple sclerosis and stroke, as well as those with musculoskeletal conditions (e.g. osteoarthritis) as it provides social connectedness among participants and addresses specific impairments including pain and reduced mobility.^13^, ^14^, ^15^ There is also growing evidence that exercising in groups where social bonds are formed leads to better performance^16^ and increased engagement in exercise in people with PD.^10^, ^11^, ^17^ People with PD are vulnerable to the impact of aquatic physiotherapy on their cardiovascular and respiratory systems^12^ but research has shown that it is a safe environment for people with PD to exercise.^12^, ^18^, ^19^ Previous studies in people with PD have found that aquatic physiotherapy has a positive impact on HRQoL^19^, ^20^, ^21^, ^22^, ^23^, ^24^, ^25^; however, only three studies were randomized controlled trials with small participant numbers^19^, ^21^, ^24^ Considering people with PD are more inactive than their community dwelling counterparts, and become even less so as the disease progresses,^26^ there is a need to better understand the perceived barriers and enablers to aquatic therapy so that engagement in exercise can be maximized for best functional outcomes in this population.

Previous literature has identified that the main enablers for people with PD to participate in a short‐term aquatic physiotherapy programme include having a supportive exercise leader and social interaction with other participants, but no specific barriers to attendance have been identified.^27^ Other research in community dwelling older adults has shown that motivational factors for aquatic physiotherapy include a reduction in pain and improved health and fitness, whilst changing clothes afterwards is a large barrier to participation.^28^ Whether the same barriers apply to people with PD is unknown. It is also unclear whether all the enablers and barriers for aquatic physiotherapy specific to the PD population have been explored. Given that exercise participation in this population is low, this area warrants additional investigation. Thus, the aim of this study was to describe the HRQoL in those with moderate PD, and explore participant perceptions regarding barriers and enablers of aquatic physiotherapy as a treatment modality.

## 2 METHODS

### 2.1 Design

**FIGURE 1:**
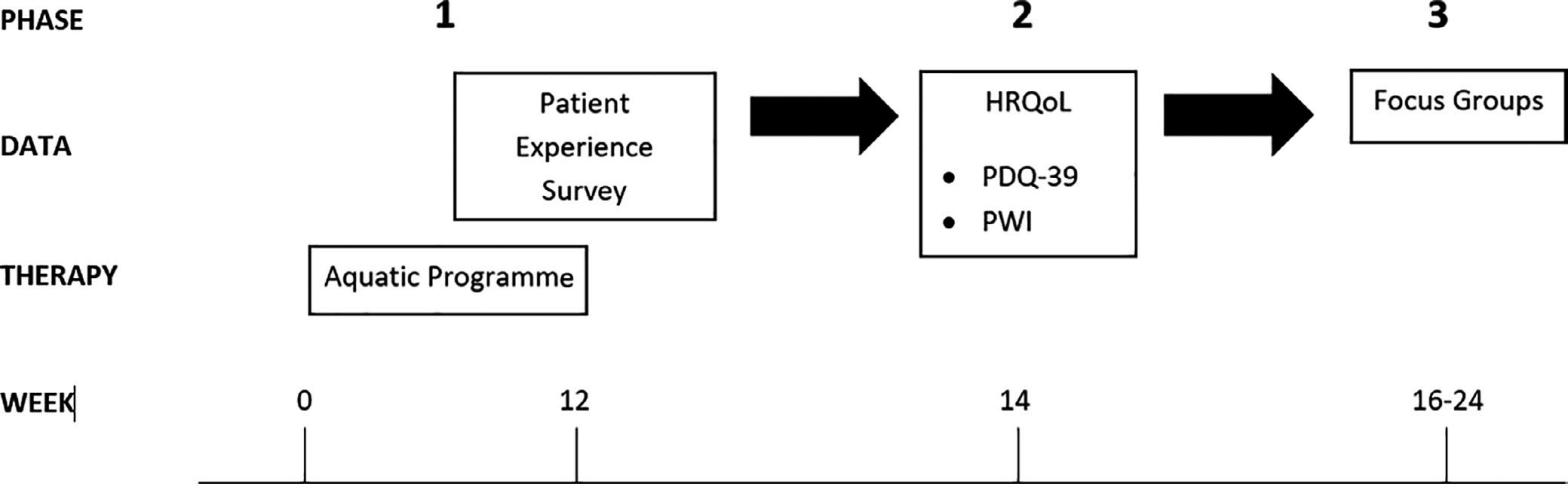
Timeline of data collection. HRQoL, health‐related quality of life; PDQ‐39, Parkinson's Disease Questionnaire‐39; and PWI, Personal Well‐being Index

This was a concurrent nested study within a previous single‐blind pilot trial (Figure 1) that examined the feasibility of a Halliwick concept style aquatic therapy programme for people with PD.^12^ Phase 1 gathered information via a survey regarding participant experiences with an aquatic physiotherapy programme. Phase 2 measured their HRQoL following completion of the aquatic programme. Finally, Phase 3 examined participants’ thoughts and beliefs about aquatic physiotherapy through focus groups. This study was completed between June 2016 and July 2018. Ethics approval was obtained from the Peninsula Health Human Research Ethics Committee (HREC) (Project 15/PH/32) and Monash University HREC (Project CF16/1341‐ 2016000731).

### 2.2 Participants

Individuals who completed the aquatic programme were invited to participate in this study. The sample and sampling procedures for the pilot trial have been described in detail elsewhere.^12^ In brief, participants were required to have a diagnosis of idiopathic PD confirmed by a neurologist, transfer and walk without assistance with or without gait aid (as clients are required to independently transfer in and out of the pool via steps), and have a Mini‐Mental State Examination (MMSE) score of 24 or above so that they can follow instructions. Participants were recruited from local Movement Disorders clinics, private neurologists and from local support groups to ensure the sample would capture the broad characteristics of people with PD. Participants were also sent information regarding the study if they had been involved previously with the Movement Disorders Program at Peninsula Health or if they had telephoned to enquire about the study.

### 2.3 Aquatic programme

Aquatic therapy was delivered by a physiotherapist and allied health assistant experienced in treating people with PD. The aquatic sessions were 60 minutes in duration, once a week for 12 weeks and were delivered in a group setting. The programme was delivered in a hydrotherapy pool (6 m × 10 m, depth 1.1‐1.5 m), with water temperature approximately 34.7 degrees Celsius and relative humidity between 63% and 76%. The pool deck temperature ranged from 25 to 31 degrees Celsius. The intervention occurred during the ‘on’ stage of the participants’ medication cycle.

### 2.4 Data collection

Data for this study included patient experience survey responses (Phase 1), measures of HRQoL (Phase 2) and focus groups (Phase 3). Demographic data such as age, gender, disease duration, disease severity as measured by the Hoehn & Yahr scale,^29^ social situation, falls history and medical co‐morbidities were also collected.

Phase 1: All participants completed an anonymous patient experience survey (Box 1) regarding their experiences about attending the 12‐week aquatic therapy programme. The survey was distributed to the participants after their last session in week 12 and participants returned the survey in a sealed box as they left.

Phase 2: HRQoL was assessed one week following completion of the aquatic therapy programme. HRQoL was quantified using the Personal Well‐being Index‐Adult (PWI)^30^ and the Parkinson's Disease Questionnaire‐39 (PDQ‐39).^31^

Phase 3: All participants were invited to attend a focus group approximately 4 weeks after completion of the aquatic programme. Semi‐structured focus groups were conducted by the primary researcher, a physiotherapist with experience in treating people with PD. An interview script with general questions (Supplementary Information 1) was used to explore participants’ perceptions.

### 2.5 Measurement instruments

The patient experience survey contained statements (Box 1) that were designed to seek feedback regarding participants’ experiences with aquatic physiotherapy. Responses to each statement were recorded using a 5‐point Likert scale that ranged from strongly disagree to strongly agree.

Box 1 **Patient experience survey statements**

Q1. I found the programme to be worthwhile

Q2. The programme was easy to follow

Q3. I felt safe at all times during exercise session

Q4. Exercises were modified to suit my limitations/ability

Q5. I enjoyed exercising with other people in a group environment

Q6. I was able to communicate with other patients and the therapist

The PWI is a measure of life satisfaction where higher scores indicate better satisfaction.^32^ The PWI consists of seven domains (standard of living, health, achieving in life, personal relationships, safety, feeling part of the community and future security) with two optional questions regarding religion and global life satisfaction. Each item is scored on a scale from 0 to 10 and converted to a number between 0 and 100. The summary index score (PWI‐SI) is an average of all domains and expressed from 0 to 100.^30^ The PWI has been shown to have good convergent validity and test‐retest reliability.^33^ Although the PWI is not a PD‐specific outcome measure, it has been used to measure life satisfaction in other neurological subgroups^34^ and comparisons with community norms can be made.

The PDQ‐39 is a disease‐specific measure of HRQoL, where higher scores indicate poorer perceived HRQoL.^31^ The PDQ‐39 consists of 39 items that are organized into eight domains (mobility, activities of daily living, emotional well‐being, stigma, social support, cognition, communication and bodily discomfort). All items are scored on a 5‐point rating scale ranging from 0 (never) to 4 (always). Summary scores can be computed for each individual domain as well as the total scale to provide an indication of the overall impact of PD on HRQoL. The PDQ‐39 is a tool that has been recommended to measure HRQoL in the PD population^7^, ^35^ and has been shown to have good content and construct validity^36^, ^37^ and high test‐retest reliability.^38^, ^39^

### 2.6 Data analysis

All quantitative data were analysed using SPSS v25.0 (SPSS Inc, Chicago, Illinois). Descriptive statistics were used to summarize the demographic and clinical characteristics of all participants.

Phase 1: Responses from the patient experience survey were classified as ‘agree’ (agree/strongly agree), ‘neutral’ and ‘did not agree’ (disagree/strongly disagree) and analysed descriptively.

Phase 2: Descriptive statistics were used to summarize the scores of the individual domains and the summary index scores of the PWI and PDQ‐39. Non‐parametric statistics such as the Friedman and Wilcoxon tests were used to examine whether there were differences between the individual domains of the PWI and PDQ‐39 with Bonferroni adjustment for multiple comparisons (*P* < .0018 for both outcome measures) because of skewness. HRQoL as measured by all domains of the PWI was compared to Australian norms^32^ and another neurological group,^34^ as there are currently no other published studies that have used PWI to assess HRQoL in people with PD. The summary index (SI) of the PDQ‐39 (PDQ‐39 SI) were compared with other therapeutic Australian studies^40^, ^41^, ^42^, ^43^, ^44^, ^45^, ^46^, ^47^, ^48^, ^49^, ^50^ identified through literature searches of three online health‐related databases (PubMed, MEDLINE, CINAHL) and targeted hand searching of reference lists. Studies were included in the comparison if the following criteria were satisfied: (a) used the PDQ‐39 to quantify HRQoL following an intervention; (b) reported the means and SDs of the PDQ‐39 SI; and (c) were full papers published in English.

Phase 3: Focus groups were audio‐recorded and transcribed verbatim by an independent party. The accuracy of transcribed qualitative data was confirmed by two independent researchers, and all transcripts were member checked by all parties involved. Data from the focus groups were analysed using the deductive coding method and were guided by the COM‐B model.^51^ The COM‐B framework is part of the behaviour change wheel that identifies 3 main components when trying to understand behaviour: capability, opportunity and motivation.^51^ This model suggests that participants require these three components to successfully engage in any given behaviour. In this framework, ‘capability’ is defined as the ability to physically and psychologically engage in activities with the appropriate knowledge and skill.^51^ ‘Opportunity’ to complete the behaviour can be physical or social, and ‘motivation’ is the conscious and subconscious processes that drive someone to complete a behaviour.^51^ Each component is able to influence another in this system.^51^ Inductive coding using open, axial and thematic coding techniques was used. Initial analysis identified key codes in the data, which were then gradually and systematically organized into larger themes. Data saturation was reached by the final (fourth) focus group. All transcripts were coded by a second independent researcher to ensure no themes were missed, and any discrepancies between the coders were resolved via discussion. If necessary, a third researcher arbitrated decisions. Quotes from the focus groups were labelled with a de‐identified participant code; for example *M, 78* refers to a male participant aged 78 years. The Consolidated Criteria for Reporting Qualitative Research (COREQ) guidelines were used to ensure correct reporting of the qualitative component of this study.^52^

## 3 RESULTS

### 3.1 Participants

Twenty‐one participants with a mean age of 70 (SD 8.2) and moderate disease severity (median Hoehn & Yahr score of 3; IQR 1) participated in this study. Overall, the mean disease duration of participants was 6 years (range 1‐23) and there was an even distribution of those who reported having a fall in the past 12 months (n = 11; 52%). Five of the 21 participants (24%) lived alone. Participant demographic and HRQoL characteristics are described in Table 1.

**TABLE 1:** Demographic and HRQoL characteristics of all participants (n = 21)

	All participants (n = 21)
Age, years	70 (8.2)
Sex, n (%)	
Males	17 (81%)
Females	4 (19%)
MMSE	27.5 (1.8)
Hoehn & Yahr, median (range)	3 (1‐3)
Disease duration, years	6 (6.3)
Levodopa use, n (%)	
Yes	20 (95%)
No	1 (5%)
Living situation, n (%)	
Alone	5 (24%)
Not alone	16 (76%)
Marital status, n (%)	
Single	6 (29%)
Married	15 (71%)
Co‐morbidities^1^, n (%)	
None	2 (10%)
Genitourinary	2 (10%)
Respiratory	3 (14%)
Circulatory	9 (43%)
Musculoskeletal	13 (62%)
Neoplasms	3 (14%)
Mental	4 (19%)
Other	9 (43%)
Self‐reported falls in 12 months, n (%)	
Yes	11 (52%)
No	10 (48%)
PDQ‐39 summary index score	31.0 (13.2)
Mobility	32.6 (23.5)
ADLs	33.7 (18.1)
Emotional well‐being	32.5 (21.6)
Stigma	23.4 (22)
Social support	18.3 (19.5)
Cognitions	29.5 (23.7)
Communication	30.6 (27.8)
Bodily discomfort	36.5 (23.3)
PWI summary index score	41.6 (13.5)
Standard of living	68.1 (21.1)
Personal health	48.6 (27.1)
Achieving in life	51.4 (28.9)
Personal relationships	71 (24.7)
Personal safety	64.3 (24)
Community connectedness	61.4 (22.9)
Future security	54.3 (27.3)
General life satisfaction (optional)	60 (18.7)

Note

All values mean (SD) unless stated otherwise.

Abbreviations: ADLs, Activities of Daily Living; and PWI, Personal Well‐being Index; MMSE, Mini‐Mental State Examination; PDQ‐39, Parkinson's Disease Questionnaire‐39.

^1^ Classified according to the International Statistical Classification of Diseases and Related Health Problems (ICD‐10).

### 3.2 Phase 1: Patient experience survey

**FIGURE 2:**
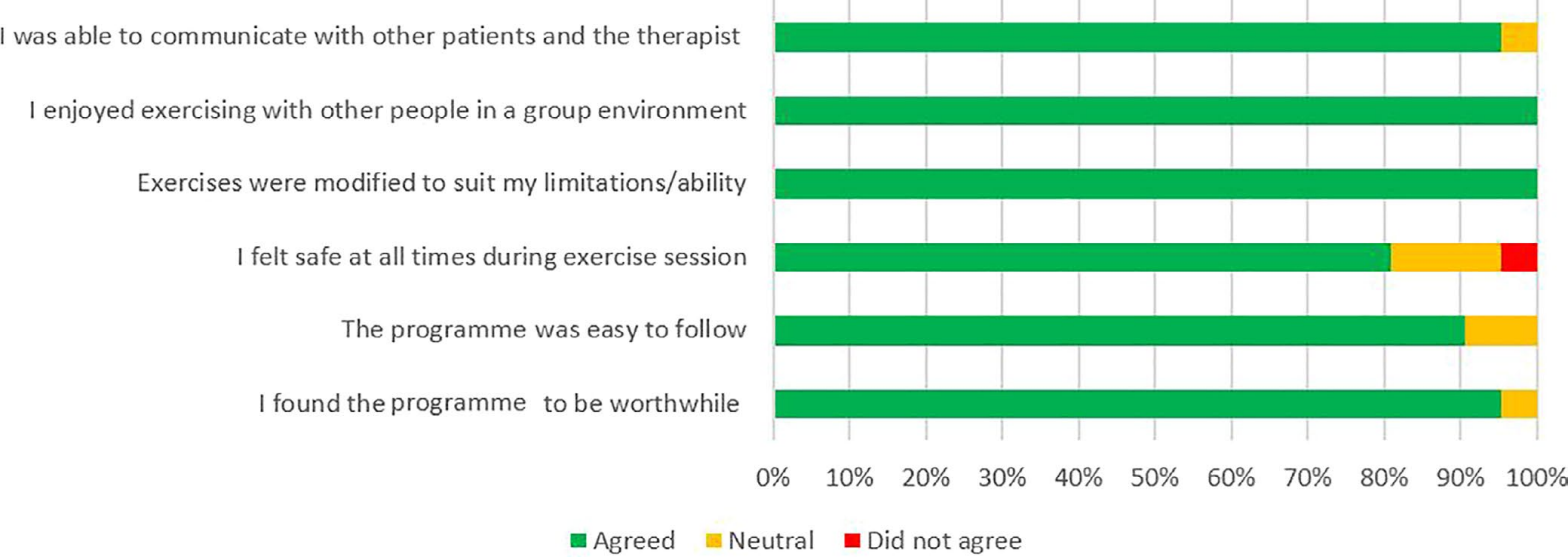
Responses to the patient experience survey

All participants (100%) completed the patient experience survey, and Figure 2 illustrates their responses. All participants reported that the exercises were adapted to suit their ability and that they enjoyed exercising with other people in a group environment. The majority felt that they found the programme to be worthwhile (n = 20/21, 95%) and that they were able to communicate with other participants and the therapist (n = 20/21, 95%). Most participants (n = 17/21, 81%) also reported they felt safe during the aquatic sessions and they felt the programme was easy to follow (n = 19, 90%).

### 3.3 Phase 2: HRQoL

**FIGURE 3:**
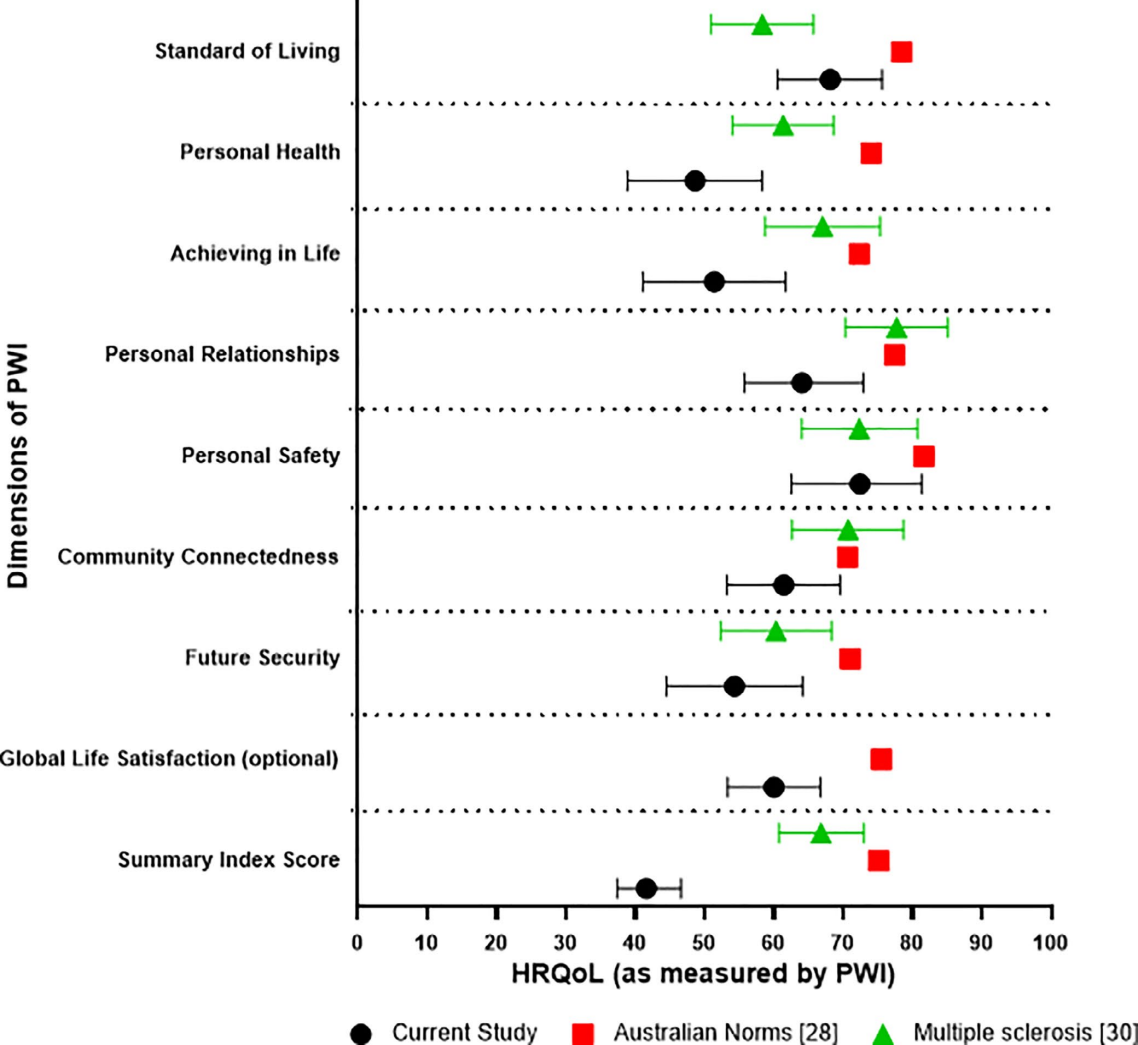
Means and 95% confidence intervals of well‐being as measured by the PWI for people with PD in this study (in black), Australian general population (in red) and multiple sclerosis (in green)

The HRQoL of participants as measured by the PWI and PDQ‐39 is summarized in Table 1. Data for the spirituality domain of the PWI were not reported as 43% of the participants (n = 9) did not respond to this optional question. Results of the PWI (Figure 3) suggest that this sample of participants had poor overall well‐being, with a mean summary well‐being score of 41.6 (SD 13.5). Participants reported that they were more satisfied with their personal safety and standard of living but were less satisfied with their personal health, life achievements and future security. A Friedman test comparing all seven PWI domains and the optional question on global life satisfaction found significant differences in the ratings across between these domains (*P* < .016). Post hoc Wilcoxon signed rank tests with a Bonferroni adjustment found participant ratings for personal health were significantly lower than ratings for personal safety (*P* < .001).

Figure 3 displays the individual domain and PWI summary index (PWI‐SI) scores for this sample of participants in comparison to Australian norms^32^ and participants with another progressive neurological diagnosis—multiple sclerosis (MS).^34^ Notably, the overall well‐being for the participants in this study was considerably lower when compared to the Australian normative data (mean 75.1, 95% CI 74.5‐75.7). Our participants were less satisfied in all domains of the PWI when compared to the overall population, with large differences observed in the domains of personal health, life achievements, personal safety, future security and global life satisfaction domains. When compared to people with MS,^34^ this sample of people with PD were less satisfied in all PWI domains, except in the standard of living domain. This sample of participants also had worse self‐perceived overall well‐being (mean 41.6; SD 13.5) compared to people with MS (mean 66.8; SD 17.2) according to the PWI‐SI score.

**FIGURE 4:**
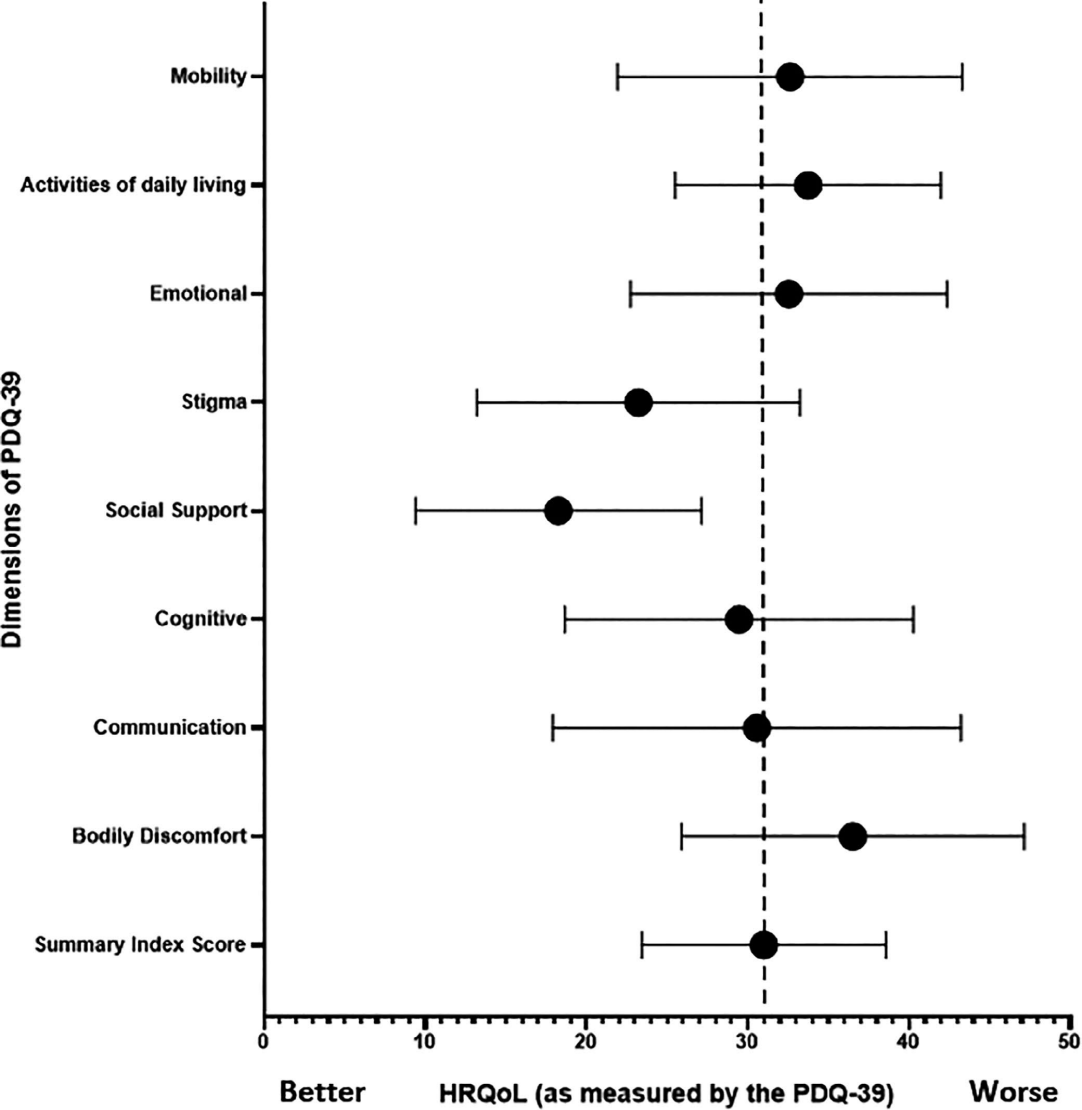
Means and 95% CI of the dimensions of HRQoL as measured by the PDQ‐39 for people with PD

The ratings for each domain of the PDQ‐39 are illustrated in Figure 4. Participants had less concerns with the domains related to social support and stigma, but reported having more difficulties in the domains of mobility, activities of daily living, emotional well‐being and bodily discomfort. No significant differences between individual domains were observed when the ratings for each dimension were compared using the Friedman test (*P* > .05 for all domains).

**FIGURE 5:**
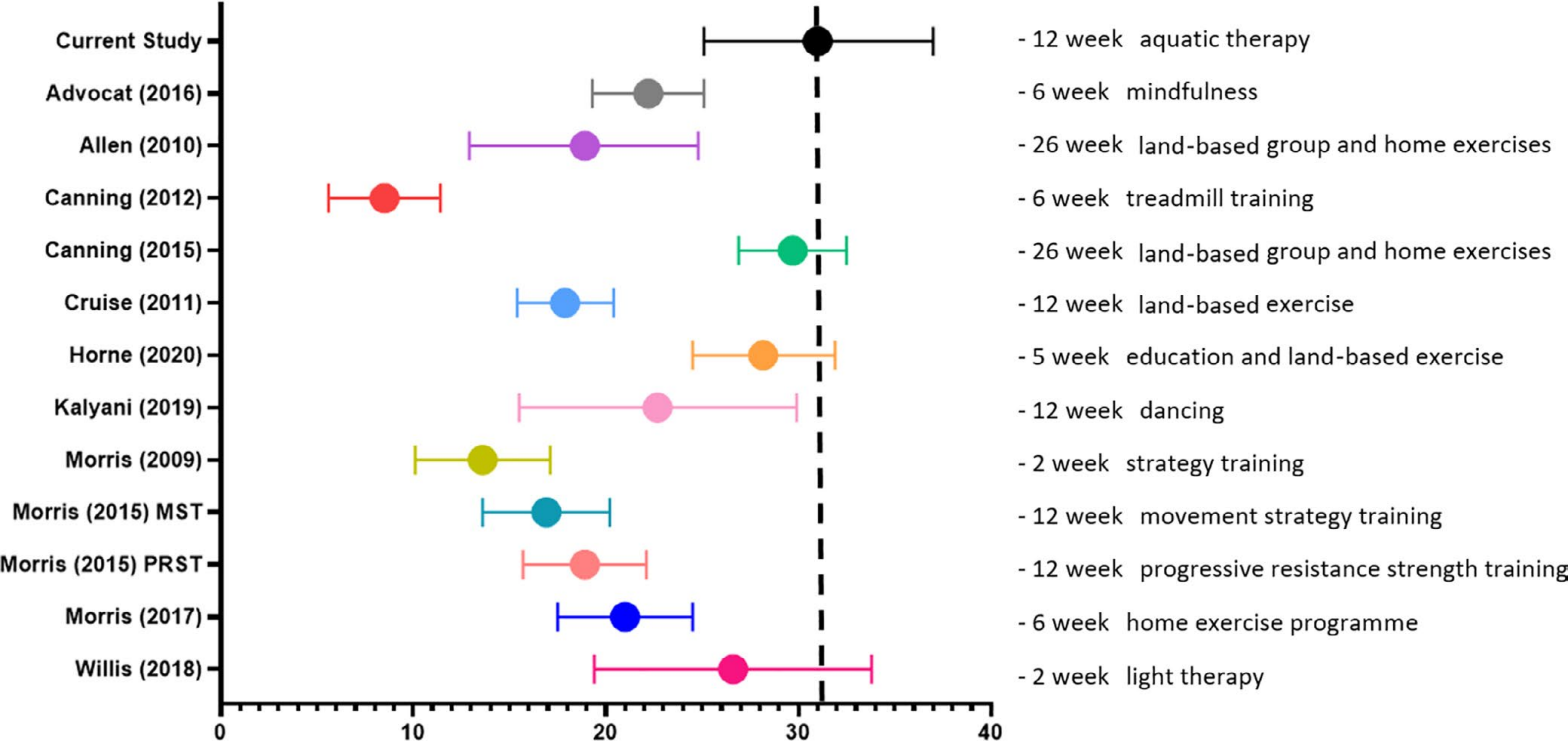
Mean and 95% CI of overall HRQoL as measured by the PDQ‐39 summary index score for people with PD living in Australia following an intervention programme

Figure 5 shows the PDQ‐39‐SI scores from this study in relation to other published Australian data reporting the HRQoL of people with PD following an intervention programme. The overall HRQoL of our sample of participants was higher (indicating poorer life quality) compared to the other studies^40^, ^53^ available for comparison.

### 3.4 Phase 3: Focus groups

Thirteen (10 males and 3 females) of 21 participants consented to take part in four separate focus groups, with groups ranging from two to four participants. Focus group participants ranged in age from 58 to 81 years (mean 73; SD 4.7) and had moderate disease severity (median Hoehn & Yahr score of 3; IQR 1). Mann‐Whitney *U* tests were performed between those who did participate and those who did not participate in the focus groups for age, disease duration and disease severity. No significant differences were found between these groups (*P* = .26). Focus groups varied in length from 40 minutes to 1 hour.

Mapping of barriers and enablers using the COM‐B framework is summarized in Table 2. Several barriers were identified for the capability and opportunity domains, and enablers for all domains, but no barriers for the motivation domain were observed.

**TABLE 2:** Mapping of barriers and enablers using the COM‐B framework

COM‐B domain		Theme
Capability Psychological and physical knowledge, skills and abilities to engage in the behaviour	Barrier	Safety in change rooms
	Enabler	Reduction in falls Improvement in gait/ Bed mobility/ Sit to stand Fitness Energy
Opportunity Physical and social outside factors which make the behaviour possible	Barrier	Transport Fatigue
	Enabler	Exercising in a group/ socialization Temperature of the pool Water environment Safety in the pool Relaxing
Motivation Automatic and reflective brain processes which direct our decisions and behaviours	Enabler	Not embarrassed Equality Group support Effort in group

#### 3.4.1 Capability

Several key themes that were identified as capability enablers to aquatic physiotherapy included a reduction in falls and improvements in function. Such functional improvements were reported in bed mobility, chair transfers and gait. One participant found that their balance was better ‘*I only know now that I don't trip over as easily’ (M, 74)*, whilst another stated that they had easier bed and chair transfers ‘*I found that I had significant benefits ….especially in how I moved around bed and stood up’ (F, 71)*. Some participants also felt that their endurance improved after the intervention ‘*I'm walking longer and further with the dog’ (M, 81)*.

Key themes that were found to be capability barriers included safety in the change rooms whilst getting dressed after getting out of the pool ‘*One of the hardest thing about the exercises was getting changed into your clothes out of your bathers’ (F, 71); ‘Getting dressed afterwards… even threading my legs into the right hole was hard’ (F, 84); ‘It was more difficult getting the clothes on than off’ (M, 61)*, and the external pool environment itself. They commented that they had to be more aware when getting out of the pool due to the water on the ground ‘*You have to be careful once you get out of the pool when going to the bathrooms as you could slip’ (M, 69)* and in the change rooms themselves ‘*I would find it hard if there were lots of people in the change rooms’ (F, 75)*.

#### 3.4.2 Opportunity

Opportunity enablers included exercising in a group (socialization), pool temperature and the water environment, and safety in the pool. Many participants felt that the pool was a better environment for exercising when compared to on land as they felt it was safer ‘*We don't have to worry about falling, so we get a full hour where our body is not handicapped’ (F, 70)* and that they were able to exercise more ‘*It was easier to do than on the ground, and more beneficial’ (M, 74)* due to the temperature of the water ‘*The warmth of the pool. It was nice and relaxing’ (F, 75)*. The majority of participants expressed they enjoyed exercising in a group environment ‘*I looked forward to being somewhere with other people’ (M, 61),* ‘*You're in a group situation, I feel greater benefit, rather than when alone’ (M, 69)*.

#### 3.4.3 Motivation

No barriers related to motivation were identified; however, several themes regarding enablers arose. This included a feeling of equality and support from the group ‘*You can laugh at each other…. You are equal so you don't feel alone’ (M, 58); ‘Everyone accepts each other for their lack of abilities, no one is picking on us, no one is different’ (F, 78); ‘You support each other when doing the exercises, you don't feel alone or embarrassed… you are one’ (M, 75)*, and increased effort when exercising in a group compared to by themselves. Participants generally felt that exercising in a group made them feel less isolated and alone with their PD symptoms.

Participants also conveyed that the group exercise aided in relaxation ‘*There is a kind of comradeship, a kind of mental therapy… when you get into the pool you relax’ (F, 55)* and resulted in a conscious increase in effort than if they had been exercising alone ‘*You are more inclined to do it in a group, if you had to do it on your own you probably wouldn't do it all’ (M, 72)*.

#### 3.4.4 Barriers

Barriers that related to opportunity included fatigue and difficulties with transportation to sessions. Several participants stated that they felt more fatigued after the aquatic physiotherapy ‘*I was more tired on the days that I did the exercise’ (F, 71)* but that this did not impact on their ability to perform their day to day tasks ‘*You do feel tired and you need a couple of hours to get over it’ (M, 78)*.

## 4 DISCUSSION

This is the first study that has used the PWI to assess HRQoL in people with PD and it provides an insight into how satisfied this cohort of people with PD are with their personal life and social situation. When compared to Australian norms, the participants in this study were less satisfied in most domains, which is not unexpected given that that the negative impact of PD on HRQoL is well‐established [11]. Lower scores for the PWI domains were also observed for this sample compared to a sample with multiple sclerosis, including their overall well‐being. This suggests that PD may have a greater impact on well‐being compared to other neurodegenerative conditions. Whilst variations in age, diagnosis, disease severity and psychosocial factors may explain these differences, further qualitative studies are needed to explore why people with PD have poorer HRQoL compared to other progressive neurological populations and examine therapies that may improve their life quality.

When comparing to other studies in Australia that have reported the PDQ‐39 SI, our participants had poorer HRQoL. This may be because participants in this study had more advanced disease (Hoehn & Yahr median 3). It is well known that HRQoL is negatively associated with disease severity^54^, ^55^ and people with PD who have greater disease disability or severity are more likely to have poor HRQoL.^1^, ^56^ In addition, there was a wide range of intervention dosage across the comparator studies ranging from 2 weeks through to 26 weeks. There was also variation in the type of intervention applied limiting direct comparisons. Whilst the differences between interventions and dosage may account for the variation in HRQoL, there may be other factors that may have contributed to life quality, such as depression and gait impairment^7^ which were not explored in this study. To our knowledge, there are no other Australian studies that have measured HRQoL using the PDQ‐39 following an aquatic intervention, with only a small number (n = 5)^20^, ^21^, ^22^, ^23^, ^57^ of studies internationally having done so. Future aquatic physiotherapy studies should consider using the PDQ‐39 to allow data to be pooled and help ascertain whether aquatic interventions have a positive impact on HRQoL in the PD population.

Responses to the patient experience survey showed that participants felt the aquatic programme was beneficial, which suggests that this may be an acceptable treatment option in the PD population. Participants in the aquatic physiotherapy programme also stated they felt they were able to communicate with others and that exercising in a group was beneficial. This is consistent with previous research that has shown that group exercises may improve engagement and participation levels, performance and motivation.^16^, ^17^ A feeling of social connectedness is extremely important in the PD population,^3^ as research has shown that as the disease progresses, so does the risk of social isolation^58^ due to feelings of embarrassment regarding the motor symptoms.^59^ Social connectedness is therefore a motivator to continue exercising in people with PD as social relationships with family and friends are an essential part of HRQoL.^3^ Participants identified that exercising with other individuals with the same condition made them feel less embarrassed*,* which again aids in social connectedness and engagement. Clinically this means that people with PD should be encouraged to exercise in groups, and in particular, in groups with other people with PD.

Prior literature has found that people with PD have low outcome expectations for exercise and that a fear of falling makes them less likely to engage in a physiotherapy programme.^9^ The results of our focus groups indicate that the majority of participants felt safe in the aquatic environment*;* therefore, this treatment modality may be more acceptable for the PD population. This adds to previous literature which has shown that aquatic physiotherapy is a safe and feasible treatment option.^12^ Although participants felt that aquatic physiotherapy resulted in improvements in balance and general function, they identified that changing from wet swimwear after the class was a barrier. Subsequently in a clinical setting, it would be beneficial to allow carers to be present to assist with doffing swimwear after exercising and/or modifying the physical environment in the change rooms (e.g. making sure height adjustable seating is available). An opportunity barrier identified was that participants felt more fatigued after the aquatic programme, but this did not stop them from completing any activities of daily living. When prescribing aquatic physiotherapy interventions, it is important to note that fatigue may impact on engagement; therefore, education regarding timing of exercise when participants are in the ‘on’ stage of their medication cycle and pacing strategies is essential.

### 4.1 Limitations

A number of limitations need noting, in particular the small sample size and cross‐sectional nature of this study. Our cohort had mild‐to‐moderate disease severity; therefore, it is unknown whether the results of this study translate to those with severe PD. We also only evaluated the experience of participants at a single facility in metropolitan Melbourne, Australia. In addition, although two‐thirds of participants attended the focus groups, the experiences of those who did not attend may not be represented in our qualitative data. Nevertheless, we utilized member checking of transcripts to ensure that the data were trustworthy. Whilst the dosage of aquatic therapy in this study did not adhere to the European Parkinson's Disease Physiotherapy Guidelines,^60^ the aim was not to determine efficacy of therapy. Our focus was to explore perceptions of aquatic therapy and to quantify the HRQoL of participants. Future larger scaled studies should consider using the TIDieR framework^61^ when reporting aquatic interventions and examine whether aquatic physiotherapy impacts on life quality.

## 5 CONCLUSIONS

Aquatic physiotherapy is a well‐accepted form of treatment in PD participants, with minimal identified barriers. Participants felt that they improved in several areas of function and that the water was a safe environment in which to exercise. Social connections made in a group exercise environment aided in the participants’ experience, and was a large enabler for therapy. Self‐perceived HRQoL is lower in individuals with PD when compared to other neurological conditions and Australian norms, suggesting that people with PD are a particularly vulnerable population that need close monitoring. This information will provide further insights into the barriers to engaging in therapy in this population, assist in modifying perceptions towards exercise, and potentially improving exercise adherence.

## CONFLICT OF INTEREST

The author(s) declare(s) that there are no conflicts of interest.

## Supporting information

Supplementary Material1

Supplementary Material2

## Data Availability

The data that support the findings of this study are available on request from the corresponding author. The data are not publicly available due to privacy or ethical restrictions.
