# Supplementary material for: Perceptions of aquatic physiotherapy and health‐related quality of life among people with Parkinson’s disease
Source: Health Expect. 2021 Feb 16;24(2):566–77. doi: 10.1111/hex.13202 (PMC8077086; doi:10.1111/hex.13202)
Supplement: Supplementary file 1 — Supplementary Material1 [file HEX-24-566-s002.docx]

**Interview Guide**

*Introduction*

The aim of today is to have a discussion amongst yourselves about the pool exercises that we did, and the importance of that, the reason why we are doing this is to gather a bit more information about how you felt doing the exercises; Whether you thought that they were of benefit and that will help us guide our future studies and will help us to decide whether we want to make those exercises part of our program here at Peninsula Health. I want you to be talking to each other if possible. If one person is talking too much I might ask them to pause for a second, and if someone is not talking very much I might ask them a question directly.

*Questions*

What did you think about your experiences here? Why?

Do you find that the exercises help or make you worse in any way? How?

Did anyone have any concerns about the program they did? Why?

Is there anything else you found challenging about the program? Why?

Do you think an hour of exercise in the pool was enough at one time? Why?
