# Supplementary material for: Perceptions of aquatic physiotherapy and health‐related quality of life among people with Parkinson’s disease
Source: Health Expect. 2021 Feb 16;24(2):566–77. doi: 10.1111/hex.13202 (PMC8077086; doi:10.1111/hex.13202)
Supplement: Supplementary file 2 — Supplementary Material2 [file HEX-24-566-s001.docx]

Supplementary information 2: Intervention characteristics in the pilot trial, taken verbatim from the published article:

Terrens AF, Soh S-E, Morgan P. The safety and feasibility of a Halliwick style of aquatic physiotherapy for falls and balance dysfunction in people with Parkinson's Disease: A single blind pilot trial. *PLOS ONE*. 2020;15(7):e0236391. doi:10.1371/journal.pone.0236391

# Participants

Participants were required to have a diagnosis of idiopathic PD confirmed by a neurologist, transfer and walk without assistance with or without gait aid (as participants are required to independently transfer in and out of the pool via steps), and have a Mini Mental State Exam (MMSE) score of 24 or above so that they can follow instructions. Those with unstable medical conditions or a self-reported history of any musculoskeletal, cardiothoracic, other neurological or psychological condition that might potentially affect participation were excluded. If there were any doubt as to medical stability, the participant’s local doctor provided a signed medical assessment form deeming suitability for aquatic and land based physiotherapy. Participants were recruited from local Movement Disorders clinics, private neurologists and from local support groups to ensure the sample would capture the broad characteristics of people with PD. There were no restrictions on disease severity, disease duration or how sedentary participants were. Participants were also sent information regarding the study if they had been involved previously with the Movement Disorders Program at Peninsula Health or if they had telephoned to enquire about the study. A sample size calculation based on a 60% recruitment rate of all participants approached [25], allowing for an attrition rate of 20%, a precision estimate of 20% and 95% confidence interval (CI), resulted in a minimum sample size of 28 participants required for this study. A target sample of 36 was established to aim for equal numbers of participants in the intervention groups.

# Procedure

Participants who expressed interest were screened by telephone, and attended the initial assessment where the blinded assessor confirmed study eligibility and obtained informed consent. Initial testing consisted of cognitive and aquatic safety screening to ensure participants were cognitively and medically suitable for study participation. Block randomisation was used for group allocation, with randomisation order developed by hand, via a third party unrelated to the study. Researchers opened an opaque, sealed and numbered envelope which determined the next intervention group. Following the initial assessment, the participant was given a sealed envelope, prepared by a research assistant, containing their allocated intervention group. There was a maximum of six participants per group, and two cohorts per intervention type. Randomisation determined the order of intervention groups, as only one group ran at a time. Details regarding demographic and clinical characteristics of participants were obtained at baseline testing.

# Interventions

All interventions were delivered by a physiotherapist and allied health assistant experienced in treating people with PD. All interventions were of 60 minutes duration per week for 12 weeks. Pre-intervention testing by the blinded assessor was undertaken one week prior to commencement of the intervention, and follow up post-intervention testing by the blinded assessor occurred one-week after conclusion of the intervention. When participants were on oral medication for their PD, testing and intervention occurred during the ”on” stage of their medication cycle.

There were three intervention groups: (1) Halliwick aquatic exercises (core specific exercises and exercises from the Halliwick concept); (2) traditional or current practise aquatic physiotherapy exercises; and (3) land based exercises (control group). Both aquatic interventions took place in a hydrotherapy pool (6m x 10m), with a depth ranging from 1.1m to 1.5m. The water temperature was approximately 34.7 degrees Celsius for all aquatic interventions, with relative humidity ranging from 63% to 76% and pool deck temperature ranging from 25 to 31 degrees Celsius. The characteristics of the exercises delivered in the three intervention groups are described in Table 1, with in depth detail of each intervention shown in Supplementary Material Table 1.

**Table 1. Intervention characteristics**

|  | **INTERVENTION GROUP** | | |
| --- | --- | --- | --- |
|  | *Halliwick Aquatic* | *Traditional Aquatic* | *Land Based* |
| Walking | ✓ | ✓ | ✓ |
| Lower Limb | × | ✓ | ✓ |
| Upper Limb | × | ✓ | ✓ |
| Strength | × | ✓ | ✓ |
| Aerobic | × | ✓ | ✓ |
| Balance | ✓ | ✓ | ✓ |
| Trunk Mobility | ✓ | ✓ | ✓ |
| Complex Rotations (sagittal, transverse, longitudinal) | ✓ | × | × |
| Core Stabilisation | ✓ | × | × |
| Stretching | ✓ | ✓ | ✓ |

All intervention groups completed walking exercises as a warm up and both upper and lower limb stretching as a cool down. The Halliwick aquatic intervention group completed trunk mobility, core stabilisation and rotational exercises as the primary exercises whereas the other intervention groups completed a range of strength, balance and aerobic exercises. The Halliwick aquatic group followed the Halliwick concept, with participants progressing through each of the 10 steps as able combined with core specific exercises. Most notably, the Halliwick concept contains complex rotational movements where the participant is fully supported by the water, and subsequently require significant core control. The trunk mobility subsection has two standing exercises, where participants’ feet are fixed on the ground. Land based exercises were matched with the traditional aquatic intervention exercises as much as possible in terms of the number of balance and cardiorespiratory exercises, types of stretches and muscle groups targeted. Exercise intensity was measured using the Borg rating of perceived exertion scale [26] during each exercise. Participants were advised to exercise to a level 13-14 on the Borg rating scale, which indicates that they were working somewhat hard in intensity. Supplementary Table 1 outlines the exercises and progressions in detail, with each participant only progressing if they completed the original exercise independently and safely.
